# Gastrointestinal bleeding as a result of entero-iliac fistula due to intestinal foreign body

**DOI:** 10.1016/j.amsu.2020.03.004

**Published:** 2020-04-01

**Authors:** Sayali Valiyeva, Lucia Romano, Francesco Maffione, Marco Leopardi, Aldo Victor Giordano, Loreto Lombardi, Mario Schietroma, Francesco Carlei, Antonio Giuliani

**Affiliations:** a Department of Surgery, San Salvatore Hospital. Department of Biotechnological and Applied Clinical Sciences, University of L'Aquila, Italy; b Vascular Surgery Unit, San Salvatore Hospital, L'Aquila, Italy; c Responsabile U.O.S. Radiologia Interventistica. San Salvatore Hospital, L'Aquila, Italy; d Surgical Endoscopy Unit. San Salvatore Hospital, L'Aquila, Italy

**Keywords:** Toothpick, Intestinal foreign body, Entero-iliac fistula, Intestinal perforation

## Abstract

Ingested toothpicks are a relatively rare event, but they may cause serious gut injuries and can be listed among rare causes of perforation, peritonitis, sepsis or death. Unless the foreign bodies were intentionally swallowed, many patients who ingested them fail to remember the event and they do not refer it during the medical history collection; this makes diagnosis problematic. In this work, a case of perforation of the sigmoid colon is described, caused by a toothpick ingestion. The patient had to be surgically treated because of a complication: the formation of an entero-iliac fistula with subsequent development of a pseudoaneurysm of the right external iliac artery. Vascular perforation due to toothpick ingestion has rarely been reported. In similar cases, it could be difficult to establishing the correct diagnosis because of the low sensitivity and accuracy rates of diagnostic investigations. The ingestion of foreign bodies should be kept in mind as an important differential diagnosis in patients with acute abdomen or chronic abdominal pain of unknown origin.

## Highlights

• Ingested toothpicks are a relatively rare event.
• These can be listed among rare causes of perforation, peritonitis, sepsis or death.
• It could be difficult to establishing the correct diagnosis because of the low sensitivity of diagnostic investigations.
• Vascular perforation due to toothpick ingestion has rarely been reported.
• The ingestion of foreign bodies should be kept in mind as an important differential diagnosis.

## 1 Introduction

Ingested toothpicks are relatively rare events, but they may cause serious gut injuries and can be listed among rare causes of perforation, peritonitis, sepsis, or death [[1], [2], [3], [4]]. Most foreign bodies pass the digestive tract freely, whereas in 10–20% of cases an interventional procedure is required. In 1% of cases an intestinal perforation occurs [5]. The ingestion of foreign bodies should be kept in mind as an important differential diagnosis in patients with acute abdomen or chronic abdominal pain of unknown origin [6,7]. The symptoms of perforation can range from relapsing blunt abdominal pain to typical peritonitis, mainly depending on the location of the lesion [8,9]. A rapid and accurate diagnosis is necessary to avoid severe outcomes [10]. In this work, a case of perforation of the sigmoid colon is described, caused by a toothpick ingestion. In similar cases, it could be difficult to establishing the correct diagnosis because of the low sensitivity and accuracy rates of diagnostic investigations [11]. Vascular perforation due to toothpick ingestion has rarely been reported [12]. This work has been reported in line with the SCARE criteria [13].

## 2 Presentation of case

**Fig. 1:**
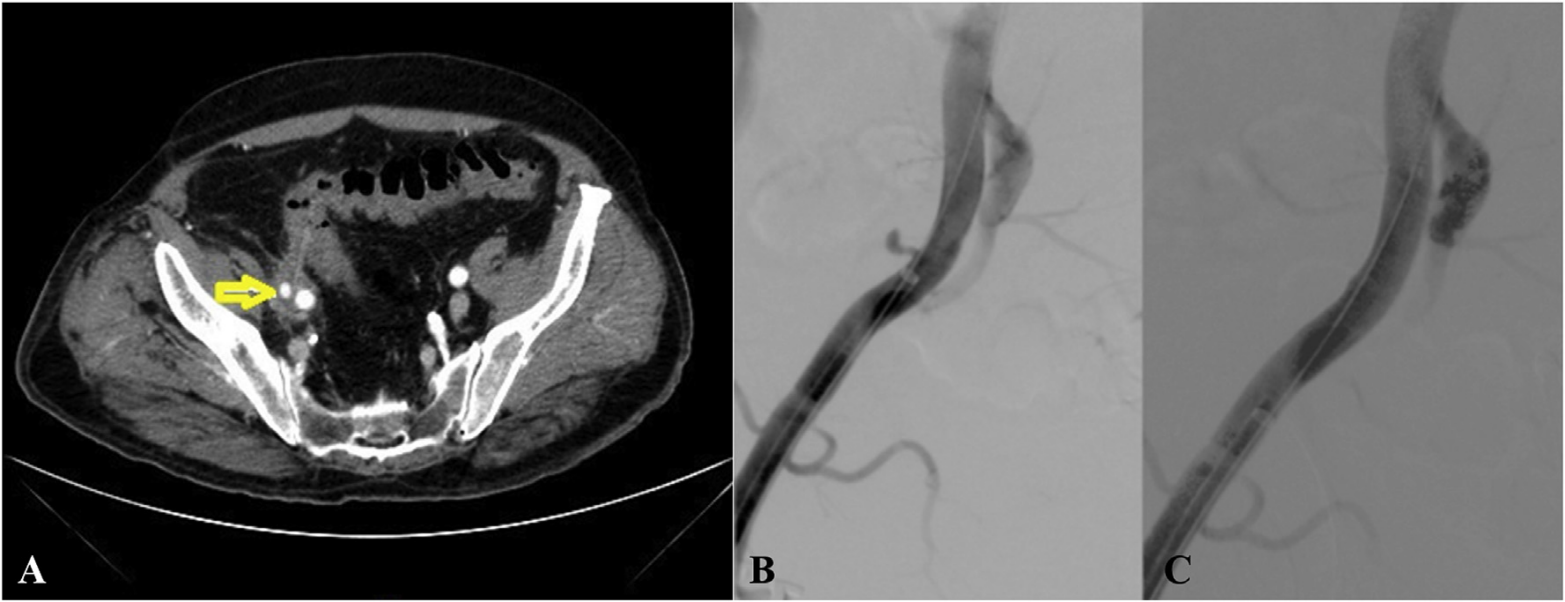
A: CT Angio-scan: a roundish formation of about 1 cm with intense arterial enhancement, referable in first hypothesis to a pseudoaneurysm of the right external iliac artery. B: Intraoperative angiography showing external iliac pseudoaneurysm. C: Completing angiography with correct stent positioning and exclusion of the pseudoaneurysm.

A 77-year-old man presented to the emergency department with two-days history of lower abdominal pain followed by rectal bleeding. His personal history revealed a stage 1 chronic kidney disease (CKD) and the presence of a heart arrhythmias treated with an implantable device. No other relevant information was reported. Laboratory testing revealed white blood count of 9,81 cells/mm3, a haemoglobin count of 10,6 g/dL and C-reactive protein value of 1,45 mg/dl; creatinine level was 1,62 mg/dl. Others laboratory tests did not report specific findings. The patient's clinical symptoms were lower abdominal tenderness and digital rectal examination (DRE) positive for blood. He underwent abdominal x-ray and US, which just highlighted distention of small intestine and transverse colon. Then, the computed tomography scan (CT) confirmed intestinal distention and also reported the presence of sigmoid diverticular formations with wall thickening of distal sigmoid loops. An area of inhomogeneous thickening with fluid density was visible between the anti-mesenteric site of sigma, external iliac artery and iliopsoas muscle, suggesting a diagnosis of diverticulitis/peri-diverticulitis. With this suspected diagnosis, patient was recovered, and a fluid and antibiotic therapy was set up. On the 4th day after recovery the patient presented an important gastrointestinal bleeding (Hb 5,5 g/dL). He was transfused and underwent an urgent CT Angio-scan, that confirmed the presence of fluid-like density alterations in correspondence to the right external iliac artery and in close proximity of anti-mesenteric site of sigma, as already reported in the previous CT exam. Moreover, it was reported the presence of roundish formation of about 1 cm with intense arterial enhancement referable in first hypothesis to a pseudoaneurysm of the right external iliac artery of unknown origin (Fig. 1A). Decision was taken to perform integration with angiographic examination for catheterization with a common ipsilateral trans-artery femoral approach, which confirmed the presence of a pseudo-aneurysmal formation. A vascular covered stent (GORE® VIABAHN®) was positioned and an intrastent angioplasty was performed to facilitate prosthetic adhesion (Fig. 1B and C). The clinical conditions improved until day 4 after procedure, when the patient became feverish (38 °C).

**Fig. 2:**
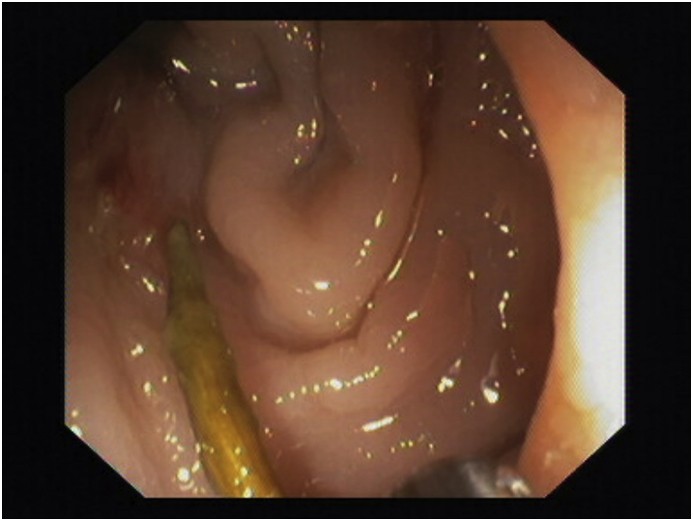
Colonoscopy image of toothpick.

A recto-sigmoidoscopy was performed, and it was diagnostic for the presence of ingested toothpick which caused the entero-iliac fistula (Fig. 2). The toothpick was removed and three hemoclips were positioned to close the internal fistulous orifice.

**Fig. 3:**
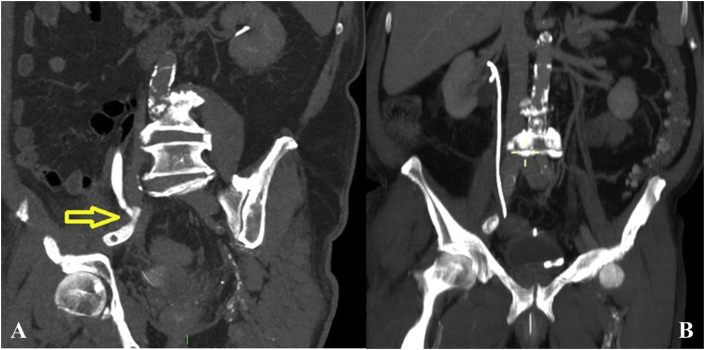
A: CT scan that evidence the distal right sided ureteral obstruction in correspondence of right external iliac artery. B: The double J ureteral stent positioned.

Five days after angioplasty, a control CT scan was performed, which documented a right sided hydroureteronephrosis (Fig. 3A). After performing urological consultation, a double J ureteral stent was positioned (Fig. 3B).

Despite of medical therapy and procedures, the patient still had fever (>38 °C) (white blood count 6.11 cells/mm3, haemoglobin value 9.0 g/dL, C-reactive protein 2.30 mg/dl, procalcitonin level 1.74 ng/ml and creatinine level 0.59 mg/dl). An urgent exploratory laparotomy was performed (A.G. and L.P.), and it was found a right iliac artery inflammation due to infected vascular endoprosthesis, with millimetric serous defect on the adjacent part of sigma. A femoro-femoral bypass and a direct closure of the sigmoid wall lesion were performed. The patient conditions gradually improved, and he was discharged on postoperative day X, with indications for post-recovery vascular surgery consultation and urological controls. Oral antibiotic therapy was suggested for ten days more. The patient was weekly followed on an outpatient basis, and his clinical conditions remained good. His last instrumental (TC scan) follow-up at 3 months did not reveal any particular problems, allowing to program the removal of the ureteral stent.

## 3 Discussion

Toothpick ingestion is a relatively rare event that can cause serious gastrointestinal injuries like perforations, bleedings, peritonitis, sepsis and even death. Unless the foreign bodies were intentionally swallowed, many patients who ingested them fail to remember the event and they do not refer it during the medical history collection; this makes diagnosis problematic [11].

In the case reported, the perforation site was the sigmoid colon. It was difficult to establish the correct diagnosis of intestinal perforation caused by toothpick ingestion because of the low sensitivity and accuracy rate of diagnostic investigations. In particular, the accuracy of CT scan to detect an ingested toothpick it is only 42,6% [1]; in fact, in our case it was just useful to detect the presence of extra-intestinal spreading of contrast medium.

The patients did not have any symptoms suggesting a peritonitis, but he had to be surgically treated because of another complication: the formation of an entero-iliac fistula with subsequent development of a pseudoaneurysm of the right external iliac artery. Otherwise, for patients who do not have complications or symptoms of peritonitis, the endoscopic removal of toothpick has proven to be successful [14].

## 4 Conclusion

The ingestion of foreign bodies should be kept in mind as an important differential diagnosis in patients with acute abdomen or chronic abdominal pain of unknown origin. We want to share our experience about this because, although rare, in these cases a rapid and accurate diagnosis is necessary to avoid severe outcomes.

## Ethical approval

Ethical approval was not required.

## Sources of funding

No funding.

## Author contribution

Sayali Valiyeva, Lucia Romano: Writing.

Francesco Maffione: Data collection.

Marco Leopardi, Aldo Victor Giordano, Loreto Lombardi: Images and contribution to the text.

Mario Schietroma, Francesco Carlei, Antonio Giuliani: Study design and review.

## Trial registry number

1. Name of the registry:

2. Unique Identifying number or registration ID:

3. Hyperlink to your specific registration (must be publicly accessible and will be checked):

## Guarantor

Dott. Antonio Giuliani.

## Provenance and peer review

Not commissioned, externally peer reviewed.

## Consent

Written informed consent was obtained from the patient for publication of this case report and accompanying images. A copy of the written consent is available for review by the Editor-in-Chief of this journal on request.

## Declaration of competing interest

No conflict of interest.
